# Effect of a bioactive pit and fissure sealant on demineralized human enamel: in vitro study

**DOI:** 10.1186/s12903-022-02617-0

**Published:** 2022-12-03

**Authors:** Rehab Samir Salma, Omnia M. AbdElfatah

**Affiliations:** 1 Pediatric and Community Dentistry Department, Faculty of Dentistry, Pharos University in Alexandria, Alexandria, Egypt; 2 Oral Biology Department, Faculty of Dentistry, Pharos University in Alexandria, Alexandria, Egypt

**Keywords:** Bioactive, EDX, Pit and fissure sealant, Remineralization

## Abstract

**Background:**

Incorporation of bioactive agent into pit and fissure sealant would halt demineralization and promote further remineralization. The aim was to assess the effect of bioactive and fluoride fissure sealants on calcium and phosphate content and surface topography of artificially demineralized enamel in young permanent teeth.

**Methods:**

30 sound extracted premolars free from cracks or any developmental anomalies were used. They were divided into group I bioactive fissure sealant, group II fluoride fissure sealant and group III no material applied. Each tooth was divided into halves in a buccolingual direction and evaluated by energy dispersive X-ray spectrometer (EDX) at baseline, demineralization and after applying the material. Another set of 7 sound extracted premolars was evaluated by scanning electron microscopy (SEM) at the same phases.

**Results:**

EDX showed that regaining calcium to demineralized enamel was significantly higher with bioactive sealant than either fluoride or the control group. SEM revealed minerals deposits with formation of distinct white zone at tooth/sealant interface for both pit and fissure sealant groups. Whereas no white zone formation was detected in control group.

**Conclusions:**

Incorporating bioactive material into pit and fissure sealant through microcapsules provided better results than incorporating fluoride by enhancing the biological process of remineralization. *Clinical relevance*: The more use of bioactive pit and fissure sealant would maintain the occlusal surfaces as sound structures and decrease the need for operative procedures to restore teeth cavitation.

## Background

Dental caries is one of the most common diseases that affect many populations. It is considered as a major public health problem worldwide [1]. Caries occurs as a result to the frequent exposure of the dental hard tissues to sugar attacks which eventually would lead to enamel demineralization [1]. Enamel is the frontline in providing protection for dentin and pulp, slowing down the progression of the dental caries process with its disastrous consequences [2]. The progression to caries is continuously prevented by the balanced process of demineralization and remineralization at the enamel surface [3]. Demineralization occurs when hydroxyapatite (HA) crystals lose its minerals. Whereas remineralization offers restoration of these minerals into the crystals [2]. However, overtime, subsurface demineralization might be too extensive for enamel to “self-heal” with the possibility of cavity formation. Therefore, extrinsic assistance is needed to stop this undermining progression. Fortunately, this is achieved through the reservoir of minerals from saliva; calcium (Ca) and phosphate (P) which aids in regaining the surface enamel layer to some degree [4]. In children, although the occlusal surfaces of their teeth constitute 12.5% of total dental surfaces, 85% of total caries incidence is seen in these areas. This is attributed to the very narrow pits and fissures (about 0.1 mm wide) which is not suitable for a standard toothbrushing to achieve effective cleaning, allowing food to be mechanically retained with biofilm formation [5]. Pit and fissure sealants have proved their effectiveness in the prevention or arrest of caries by providing these occlusal surfaces with a physical barrier [6]. However, the frequent use of fissure sealants showed some drawbacks. Among those, the occurrence of microleakage along the sealant’s margins with the possibility of subsequent enamel demineralization adjacent to the material at the sealant-tooth interface [7]. It is quite advantageous when the pit and fissure sealant has remineralizing properties. The fourth generation of resin-based sealants have fluoride-releasing abilities. Fluoride can enhance remineralization, stop demineralization, and increase the dissolution-resistance of the tooth structure. Nevertheless, this effect depends on the amount of fluoride that can be released [8].

Currently, biologic approaches of caries management are based on the caries risk of each individual, on the minimal non-invasive treatment methods and on the bioactivity of the utilized material [9]. Bioactive material is the one able to induce a biological effect on the host tissue. This depends on the ability of the material to induce re-precipitation of HA on enamel and dentin surfaces or to release biologically active substances and ions that have great cellular changes [10]. The available bioactive restorative materials are few, they are known for their release and recharge of ionic components in response to pH changes. They are of two types; one which could contain different kind of ion releasing glasses (e.g., Activa™, Pulpdent; Predicta™ Bioactive, Parkwell; Giomer™, Shofu) and the other type that encloses semipermeable microcapsules associated with special monomers. These microcapsules would produce better physical properties to the material combined with its bioactivity. (e.g., BioCoat™, Premier) [11]. It has been introduced as a bioactive pit and fissure sealant. It has microcapsules which are filled with ionic solutions of fluoride, calcium, and phosphate and would be rechargeable by diffusion of ions in and out of the sealant [12]. This mode of action should be investigated thoroughly because it is suggested that microcapsules release considerable quantities of ions for remineralization in biological conditions. To our knowledge, no studies were published until now to compare the remineralizing ability of a bioactive fissure sealant to a fluoride containing fissure sealant. Accordingly, the aim of this study was to assess the remineralizing effect of the bioactive sealant through its microcapsules compared to a fluoride-containing pit and fissure sealant as evidenced by the change in the mineral content and surface topography of artificially demineralized enamel surfaces in young permanent teeth. The null hypothesis was that there is no difference in remineralization effect between the bioactive pit and fissure sealant and the fluoride-containing pit and fissure sealant whether on the mineral content or the surface topography.

## Methods

This is an in vitro study, which was conducted at the Pediatric Dentistry and the Oral Biology departments, Faculty of Dentistry, Pharos University in Alexandria, Alexandria, Egypt. Ethical approval was obtained from the Research Ethics Committee—Pharos University in Alexandria in adherence to the tenets of the Declaration of Helsinki 1964 and its later amendments (# PUA02202208283038) [13]. Two sets of samples were used according to the employed evaluation technique. The first set was evaluated by energy dispersive X-ray spectrometer (EDX) and analysed for minerals content while the second set was evaluated by scanning electron microscopy (SEM). The first set of samples consisted of 30 sound extracted premolars. They were collected from patients aged 15–20 years who were scheduled for serial extraction as part of their orthodontic treatment. An informed consent to participate in the study was obtained from older participants and from parents and/or legal guardians of minor participants. This was accomplished after a thorough explanation of the purpose and aim of the study. The collected teeth were free from cracks or any developmental anomalies. All teeth were debrided, cleaned with pumice paste to remove any residual plaque or stains specially from the occlusal surfaces and then stored in distilled water to avoid enamel dehydration till we started working. The teeth were randomly divided into three groups each containing 10 teeth according to the used material. All teeth were sectioned into 2 halves in a buccolingual direction to allow studying the changes in enamel crystals along the depth of the fissure.

Roots were removed 3 mm below the cementoenamel junction using a double-sided water-cooled diamond disc at low speed. Crowns were inserted in acrylic resin block then sectioned longitudinally in a bucco-lingual direction into halves using a double-sided fine grit water-cooled diamond disc with care to avoid damage to the inner surface. Teeth specimens were about 4 mm in thickness and were polished by diamond paste of 1 μm size. Then, they were washed, dehydrated and air dried. Of each tooth, only one half was processed and evaluated by EDX at all subsequent stages: baseline, demineralization, and pH cycle. Group I included teeth that were sealed with bioactive fissure sealant (BioCoat pit and fissure sealant with bioactive ions- Premier Dental Products Company). Group II included teeth that were sealed with fluoride fissure sealant (Fisseal pit and fissure sealant containing fluoride- PROMEDICA Dental Material). Group III included teeth that were not sealed and would serve as control group.

**Fig.1:**
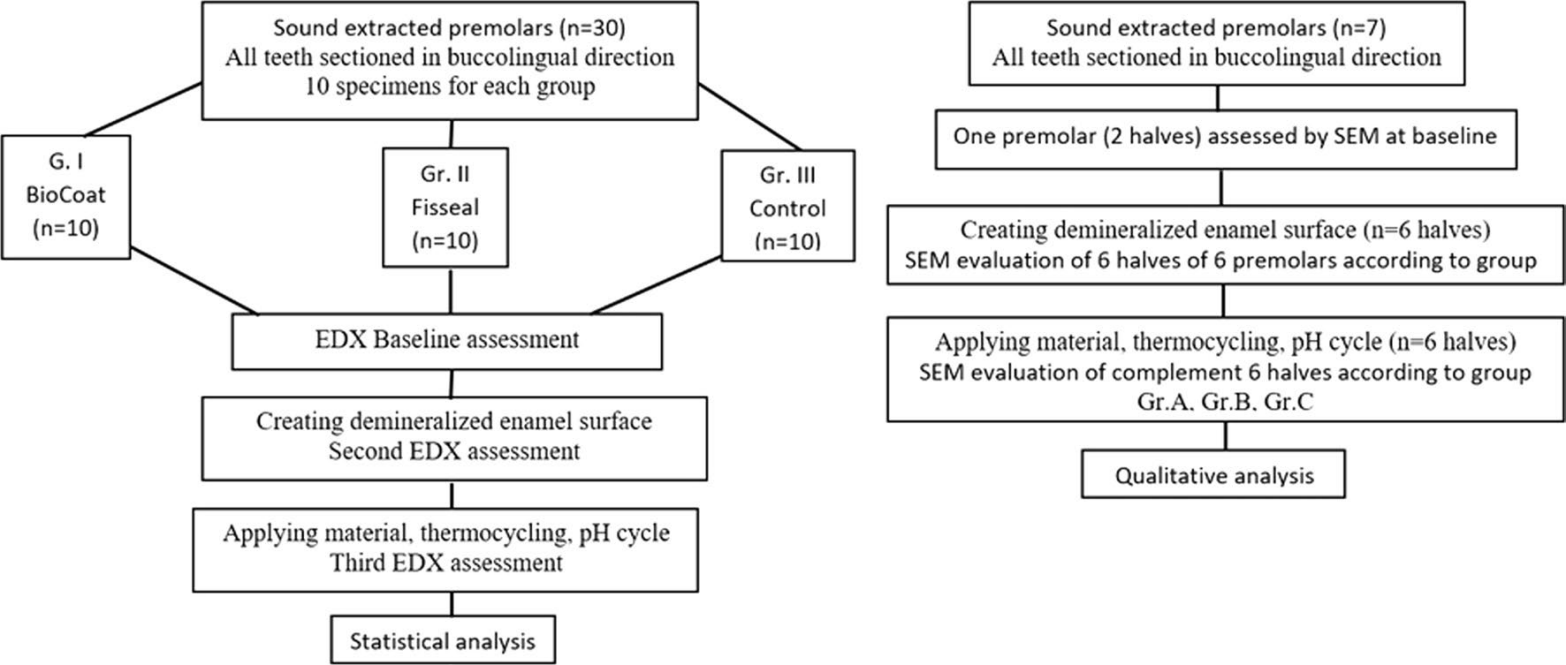
Flow chart illustrating the main steps

The second set of samples consisted of 7 sound extracted premolars. They were selected according to the same criteria used with the first set and likewise were stored in distilled water to avoid enamel dehydration till the start of work. They were divided as follows: one tooth, after being sectioned buccolingually as previously mentioned, served as baseline SEM evaluation (both halves were evaluated). While the other 6 premolars were sectioned in a buccolingual direction, and 1 half of each tooth was evaluated by SEM at the demineralization stage. The complement half served for SEM evaluation at pH cycle stage. According to the material used, 2 premolars were assigned for Gr. A (bioactive fissure sealant). Another 2 premolars were assigned to Gr. B (fluoride fissure sealant) whereas the last 2 premolars were assigned for the control group Gr. C. Figure 1 shows a flow chart illustrating the main steps of the current study.

### EDX evaluation

To assess the same surface at different intervals, we used electrically conductive carbon tapes to attach each sample to the chamber’s base. This facilitated the conductivity of surfaces without affecting the minerals under investigation. Each sample was mounted on a conductive tape onto the chamber just before analysis. The tape was facing downwards and in no contact with the examined surface. After sample removal, it was cleaned with ethanol.

#### First assessment (at baseline)

All 30 specimens of groups I, II and III were evaluated using EDX (high vacuum mode, voltage 20 kV) at the area just beneath the occlusal groove as a baseline evaluation of mineral content.

#### Second assessment (after demineralization)

Squares of self-adhesive labels 2 × 2 mm were stuck at the occlusal groove and the area just beneath it. All surfaces were then coated with nail varnish. The adhesive labels were then removed exposing a small window which included the occlusal groove and the area below it. Creating subsurface carious lesions was achieved by immersing each specimen into separate tube containing 40 ml of demineralizing solution for 96 h to achieve an artificial lesion of 120–200 μm depth. The solution composed of 2.2 mM Calcium chloride (CaCl_2_), 2.2 mM Potassium dihydrogen phosphate (KH_2_PO_4_), 0.05 M Acetic acid (CH_3_COOH), and to adjust the pH to 4.4, 1 M Potassium hydroxide KOH was added [14]. By the end of the 96 h, re-evaluation of the mineral content for each specimen in all groups was performed again at the area just beneath the occlusal groove.

#### Third assessment (after applying materials)

First, the sectioned surfaces were covered again with self-adhesive labels to prevent the dripped material of contacting those surfaces and then specimens were fixed in paraffin wax for ease of manipulation. The occlusal grooves of all specimens in all groups I, II and III were then acid etched with 35% phosphoric acid gel for 20 s, rinsed and lightly air dried. In group I, the etchant used was Premier® Etch (Premier Dental Products Company. USA). This was followed by applying bioactive fissure sealant (Premier Dental Products Company. USA), according to the manufacturer’s instructions then light cured for 20 s using Elipar™ DeepCure-S LED Curing Light (3 M Ltd., USA). In group II: the etchant used was Cica™ (PROMEDICA Dental Material GmbH, Germany). It was followed by applying fluoride fissure sealant (PROMEDICA Dental Material GmbH, Germany), according to the manufacturer’s instructions then light cured for 20 s using Elipar™ DeepCure-S LED Curing Light (3 M Ltd., USA). As for group III: the etchant used was Premier® Etch (Premier Dental Products Company. USA) but no material was applied afterwards on the occlusal grooves.

The self-adhesive labels were removed from sectioned surfaces then all specimens of all groups were placed in distilled water at 37 °C for 24 h. Then to simulate the oral environment, they were subjected to thermal fatigue through thermocycling 500 times between 5 and 55 °C with a transfer time of 10 s and a dwell time of 30 s in each water bath. An acid-resistant varnish coating was re-applied to the previously varnished surfaces leaving a small window of the occlusal groove and the area below it by using again the self-adhesive labels 2 × 2 mm. Following removal of labels, all specimens were then subjected to pH-cycling for two weeks. For this purpose, the same demineralizing solution was employed. As for the remineralizing solution, it consisted of the following components: 1.5 mM Calcium chloride (CaCl_2_), 0.9 mM Sodium dihydrogen phosphate (NaH_2_PO_4_), 0.15 M Potassium Chloride (KCl) with a pH of 7.0, 20 mmol/L cacodilate buffer. All specimens were individually suspended into tubes containing 40 ml of either solution. They were submitted to immersion in the demineralization solution for 6 h. Then they were rinsed with deionized water followed by immersion for 18 h in the remineralizing solution. This was performed for 10 days-period and after each 5 days-period, specimens were immersed in the remineralizing solution for 2 days [14]. As pH cycle ended, EDX re-evaluation of the mineral content for each specimen of all groups was performed at the area just beneath the occlusal groove. All EDX readings were compared to baseline and to subsurface demineralization readings for all groups.

### SEM evaluation

#### Preparation of specimens

All assigned specimens were allowed to dry for 24 h before subjecting them to the gold ion sputtering. Afterwards they were mounted on specimen stumps making the area to be studied facing upwards. Next, they were coated with a thin layer, of 30 μm thickness, of pure gold using an ion sputtering unit of 1000 V. These prepared specimens were placed in the vacuum chamber of the scanning electron microscope (voltage at 50 kV). The surface was evaluated on the screen under 550X magnification. The qualitative changes at the tooth surface and at the tooth/sealant interface were observed at all designated stages for all groups as previously mentioned. The presence of either granular, globular or white zones at the interface was considered positive for remineralization [15].

### Statistical methodology

Data were fed to the computer and analyzed using IBM SPSS software package version 20.0. (Armonk, NY: IBM Corp). The Kolmogorov–Smirnov test was used to verify the normality of distribution. Quantitative data were described using range (minimum and maximum), mean, standard deviation. Significance of the obtained results was judged at the 5% level. F-test (ANOVA) was used for normally distributed quantitative variables, to compare between all groups, and Post Hoc test (Tukey) (LSD) was added for pairwise comparisons. Kruskal Wallis test was used for abnormally distributed quantitative variables, to compare between all three periods regarding percentage change, and Post Hoc (Dunn's multiple comparisons test) was applied for pairwise comparisons. For each group, the percent change in minerals was calculated as follows: [(Remineralization-Demineralization)/Demineralization] *100 and the recovery percentage for all groups was calculated as follows:

$$\left[ {\left( {{\text{Remineralization}} - {\text{Demineralization}}} \right)/{{(Baseline}} - {\text{Demineralization)}}} \right]*100$$

## Results

### Energy dispersive X-ray spectrometer analysis

In Tables 1and 3, Ca content showed that after remineralization, it significantly increased in group I (bioactive sealant) than the other groups (36.18 ± 4.74, *p* < 0.001*). Group I was significantly higher than group II (36.18 ± 4.74, 32.39 ± 2.47, *p* = 0.036*) and than group III (36.18 ± 4.74, 26.62 ± 1.64, *p* < 0.001*). While group II (fluoride sealant) was significantly higher than group III (control group) (32.39 ± 2.47, 26.62 ± 1.64, *p* < 0.001*). Percentage increase of Ca from demineralization to remineralization was significantly higher in group I compared to other groups (↑59.37 ± 14.40, *p* < 0.001*). Percentage increase of Ca in group I was significantly higher than in group III (↑59.37 ± 14.40, ↑12.58 ± 5.87, *p* < 0.001*). Percentage increase of Ca in group II was significantly higher than group III (↑54.91 ± 15.85, ↑12.58 ± 5.87, *p* < 0.001*). Percentage increase of Ca was recorded for group I compared to group II although it was not significant. Ca recovery percentage showed the highest increase with group I compared to other groups (127.2 ± 64.91, *p* = 0.007*). Ca recovery percentage was significantly higher in group I than group III (127.2 ± 64.91, 60.15 ± 36.51, *p* = 0.002*). Ca recovery percentage was significantly higher in group II than group III (99.29 ± 19.51, 60.15 ± 36.51, *p* = 0.045*). Higher Ca recovery percentage was recorded for group I compared to group II although it was not significant.

**Table 1:** Calcium (Ca) and phosphate (P) content, percent change and recovery percentage within all groups, at baseline, demineralization and after remineralization

	Mineral content			Percent change	Recovery percentage mean ± SD
	Baseline mean ± SD	Demineralization mean ± SD	Remineralization mean ± SD	Demineralization to Remineralization mean ± SD	
Ca in Gr I	35.98 ± 9.24	22.83 ± 3.22	36.18 ± 4.74a,b	↑59.37 ± 14.40a	127.2 ± 64.91a
Ca in Gr II	32.82 ± 4.21	21.10 ± 2.58	32.39 ± 2.47a	↑54.91 ± 15.85a	99.29 ± 19.51a
Ca in Gr III	29.14 ± 1.57	23.67 ± 1.34	26.62 ± 1.64	↑12.58 ± 5.87	60.15 ± 36.51
Test of Sig. (p)	F = 3.338 p = 0.051	F = 2.752 p = 0.082	F = 22.231* p < 0.001*	H = 19.667* p < 0.001*	H = 9.881* p = 0.007*
P in Gr I	37.75 ± 10.40	42.81 ± 11.13	39.46 ± 3.85a	↓0.15 ± 3.27	94.41 ± 52.03
P in Gr II	41.78 ± 4.52	48.99 ± 4.92	41.36 ± 3.70a	↓14.39 ± 15.14a	937.6 ± 2553a
P in Gr III	43.68 ± 3.41	44.81 ± 3.09	47.24 ± 3.33	↑5.65 ± 7.27	-608.6 ± 1906
Test of Sig. (p)	F = 1.963 p = 0.160	F = 1.890 p = 0.171	F = 12.476* p < 0.001*	H = 7.628 p = 0.022*	H = 6.831* p = 0.033*

F: F for ANOVA test. H: H for Kruskal Wallis test

a: Statistically significant with group III

b: Statistically significant with group II

*Statistically significant at p ≤ 0.05

As regards P content, it showed after remineralization a significant increase in group III than the other groups (47.24 ± 3.33, *p* < 0.001*). Group I was significantly lower than group III (39.46 ± 3.85, 47.24 ± 3.33, *p* < 0.001*) and group II was significantly lower than group III (41.36 ± 3.70, 47.24 ± 3.33, *p* = 0.003*). Percentage decrease of P from demineralization to remineralization was significantly lower in group II compared to other groups (↓14.39 ± 15.14, *p* = 0.022*). Percentage decrease of P in group II was significantly lower than group III (↓14.39 ± 15.14, ↑5.65 ± 7.27, *p* = 0.007*) while percentage decrease of P was recorded for group I compared to group II and compared to group III although this was not significant. P recovery percentage was significantly higher in group II compared to other groups (937.6 ± 2553, *p* = 0.033*). P recovery percentage was significantly higher in group II than group III (937.6 ± 2553, −608.6 ± 1906, *p* = 0.011*) while there was no significant difference between groups I and II or groups I and III.

Tables 2 and 3 showed that Ca/P ratio was significantly higher after remineralization in group I than the other groups (0.94 ± 0.24, *p* < 0.001*). Group I was significantly higher than group III (0.94 ± 0.24, 0.57 ± 0.06, *p* < 0.001*) and also group II was significantly higher than group III (0.79 ± 0.13, 0.57 ± 0.06, *p* = 0.010*). Higher Ca/P ratio was recorded for group I compared to group II although it was not significant.

**Table 2:** Ca/P ratios within all groups, at baseline, demineralization and after remineralization

Ca/P ration	Gr I	Gr II	Gr III	F (p)
Baseline Mean ± SD	1.25 ± 1.25	0.80 ± 0.20	0.67 ± 0.07	1.685 (0.204)
Demineralization Mean ± SD	0.58 ± 0.22	0.44 ± 0.07	0.53 ± 0.06	2.864 (0.074)
Remineralization Mean ± SD	0.94 ± 0.24^a^	0.79 ± 0.13^a^	0.57 ± 0.06	13.761* (< 0.001*)

F: F for ANOVA test

a: Statistically significant with group III

*Statistically significant at p ≤ 0.05

**Table 3:** Pairwise comparisons between all groups regarding mineral content, percent change, recovery percentage and Ca/P ratios after remineralization

	Mineral Content			Percent change			Recovery percentage			Ca/P ratio		
		Gr I	Gr II		Gr I	Gr II		Gr I	Gr II		Gr I	Gr II
Ca	Gr II	0.036*		Gr II	0.576		Gr II	0.275		Gr II	0.124	
	Gr III	< 0.001*	< 0.001*	Gr III	< 0.001*	< 0.001*	Gr III	0.002*	0.045*	Gr III	< 0.001*	0.010*
P	Gr II	0.480		Gr II	0.416		Gr II	0.461				
	Gr III	< 0.001*	0.003*	Gr III	0.060	0.007*	Gr III	0.071	0.011*			

Tukey and Dunn’s Post-Hoc tests for pairwise comparisons for all groups

*Statistically significant at *p* ≤ 0.05

### Scanning electron microscopy results

**Fig. 2:**
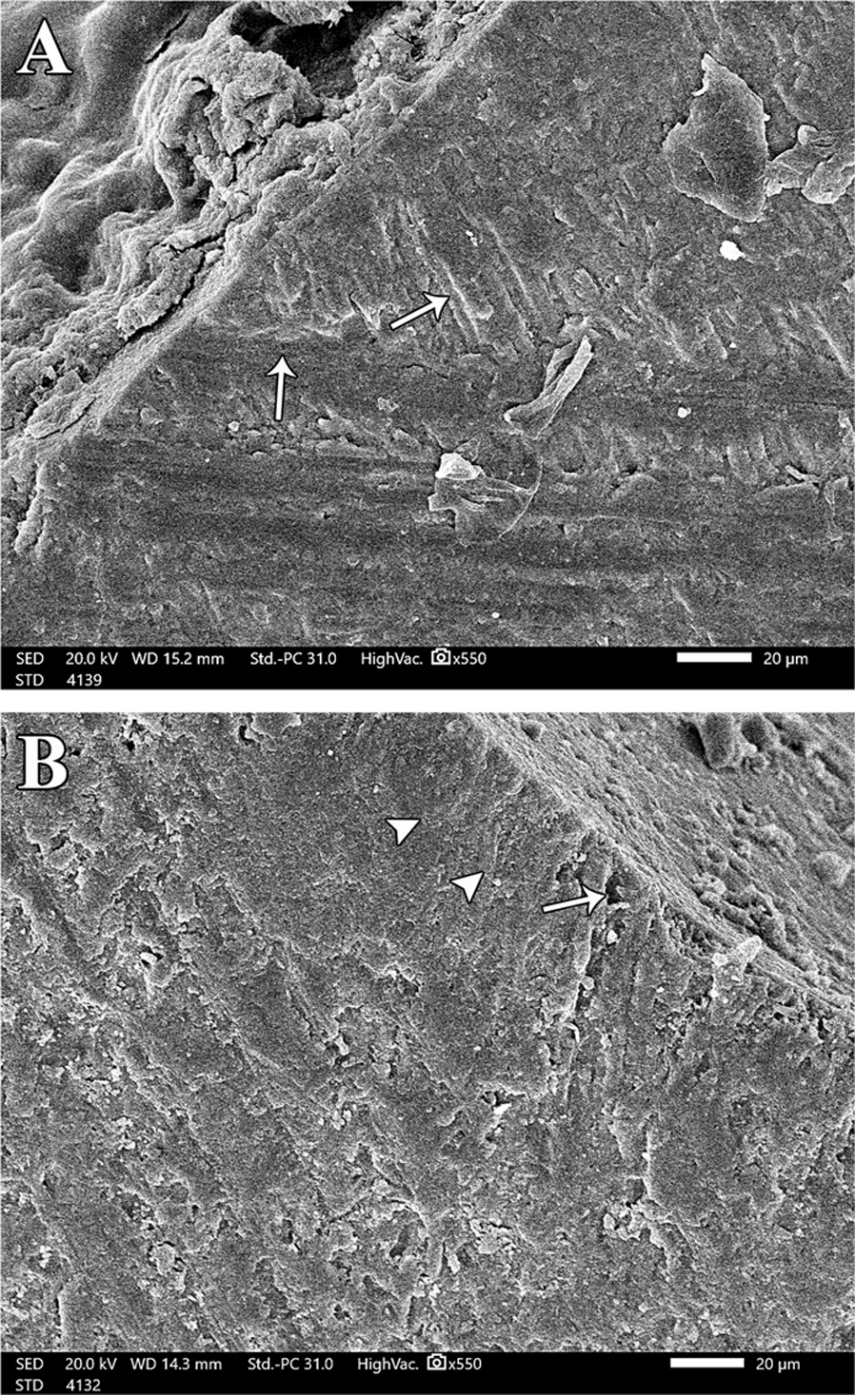
Normal and demineralized enamel structure. Two scanning electron micrographs, longitudinal section (SEM-LS) of exposed enamel surface around the central groove (magnification x 550) where (**A**) is at baseline, showing normal intact enamel structure with few minute depressions while (**B**) is after demineralization. There is evidence of collapse of enamel rods and lack of orientation and irregularity of the HA crystals and there is evidence of micro spaces on the enamel surface.

**Fig. 3:**
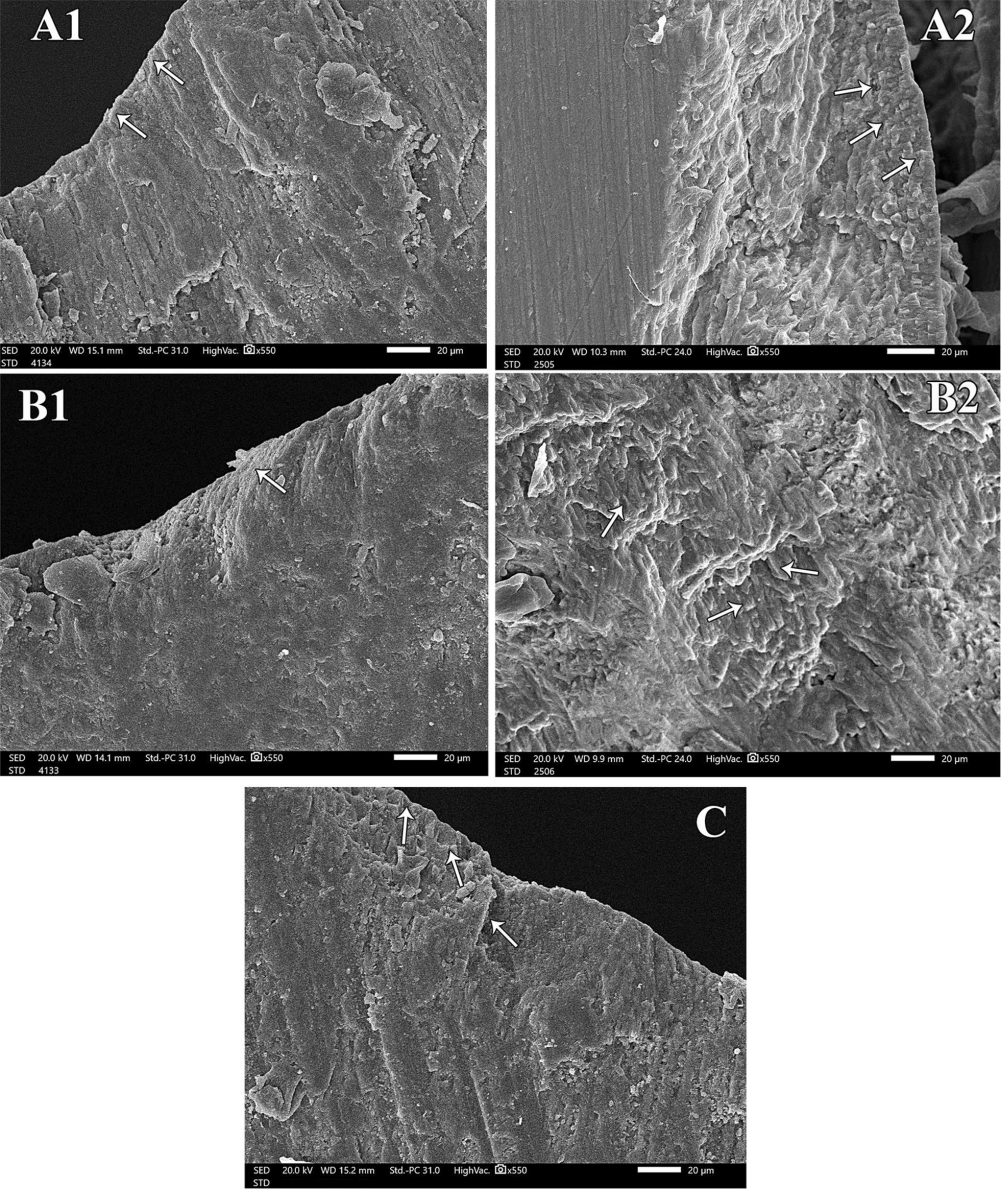
Examination of groups A, B and C after pH cycle. Five scanning electron micrographs, longitudinal section (SEM-LS) of exposed enamel surface around the central groove after pH cycle where **A-1** is presenting Gr. A magnification (x 550). The enamel surface is smooth and uniform with scarce depressions. Intact enamel surface with no observable defects can be detected. The tooth/sealant interface is showing white zone along the enamel surface. **A-2** Gr. A magnification (x 550) is showing multiple granular zones at the interface. **B-1** is showing Gr. B magnification (x 550) with minor surface irregularities and areas of defective change nearly similar to normal enamel surface characteristics. Wide white zone is observed at tooth/sealant interface. **B-2** is Gr. B magnification (x 550) revealing occasional shallow pits with apparent irregular granular zone. **C** is Gr. C magnification (x 550) showing roughness in the enamel surface with multiple pits. These pits are of varying depth and width. There are noticeable defects along the whole enamel surface. No detection of any white zones along the interface.

Changes in the enamel just beneath the central groove of all groups was assessed by SEM. At baseline, enamel surface was presented with its characteristics, yet minute depressions were spotted in the micrographs (Fig. 2A). Demineralization in all groups exhibited several defective areas on the enamel surface with evidence of collapse in enamel rods, lack of orientation and rough micro spaces scattered on the surface (Fig. 2: B). Remineralization of the decalcified enamel surface was evaluated for all groups. Group A (bioactive sealant) showed a uniform smooth intact surface. White zone along the sealant/tooth interface was noticed and there were some wide irregular granular zones at the interface representing mineral crystals (Fig. 3A-1 and A-2). Group B (fluoride sealant) exhibited minor defects and irregularities similar to the typical enamel surface characteristics. A distinctive white zone was observed at sealant/tooth interface. Occasional shallow pits with apparent irregular granular zones were detected (Fig. 3B-1 and B-2). Group C (control) showed roughness in the enamel surface with multiple pits of varying depth and width. There were noticeable defects along the whole enamel surface and no detection of any white zones along the interface. (Fig. 3C).

## Discussion

Enamel is acellular tissue which has no self ability for regeneration. Extra means for remineralization are needed to act as mediators in enhancing remineralization [3]. Although several materials are widely investigated for their enamel remineralizing potential [4], there is still a disagreement about their classification as well as their methods of application [11]. Since remineralization would not occur unless adequate amounts of Ca and P ions are available, so an ideal remineralizing agent should be bioavailable in saliva to facilitate diffusion of these ions into the caries lesion [16]. A study measured the release of ions from fissure sealants containing microencapsulated remineralizing agents. They concluded that the released Ca and P ions, from these microcapsules, played an important role in the effective incorporation of fluoride onto the enamel surface during remineralization [17]. Various investigations on nanoparticle-enhanced fissure sealants focused on the sealants' mechanical and chemical characteristics, as well as their antibacterial and remineralization properties [18].

The current study was conducted to evaluate the remineralizing effect of one type of bioactive pit and fissure sealant through its microcapsules’ technology compared to a fluoride-containing pit and fissure sealant. These microcapsules were actively participating in ionic exchange on the enamel surface which strengthens the structure of the surface and subsurface enamel. The released ions would supersaturate the oral environment around the enamel providing protection against demineralization during acid attacks [19]. Fluoride sealant was chosen as a positive control material that is already known for enhancing remineralization through its ability to release fluoride. A third group, where no material was applied to the occlusal surface, served as negative control group.

The study used an in vitro model due to its stability, low cost and to reduce the influence of confounding factors. pH cycling model was employed to mimic the oral environment periodic changes of pH that resembles the caries process [14, 20]. The outermost layer of enamel which is approximately 30 µm is highly calcified [21]. That was the reason the area designated for examination was the area beneath the central groove. This area was chosen to overcome the possible masking effect of the significantly high mineral content in the outer layer which could intervene with the artificial demineralization process and at the same time to create the artificial enamel lesion through subsurface demineralization. EDX is a chemical elemental microanalysis technique which was widely used in measuring mineral content at the ultrastructural level in several studies [22]. It was employed here to measure the remineralization ability of both materials on the demineralized enamel surface and to compare between the effect of both materials on the enamel surface. While qualitative analysis using SEM was added to observe the mineral deposits on the same demineralized surfaces.

EDX recorded the changes in Ca, P, and the Ca/P ratio, as indicators of mineral regaining ability of materials under investigation and to enhance the accuracy of the results baseline readings was recorded. The results demonstrated that bioactive sealant could regain the mineral content into the artificially decalcified enamel surface. This could be explained by the effect of continuous ions flow through the microcapsules [12]. The bioactivity of the material might have enabled the increase in Ca and P concentrations and the release of fluoride within carious lesions to a higher level than that existing in normal oral fluids allowing for a great potential to enhance remineralization [19, 23]. This is consistent with the study conducted by Burbank et al. [17]. Furthermore, the availability of fluoride needed for remineralization within the microcapsules is another reinforcing way for inhibiting further demineralization and enhancing remineralization which is in agreement with what was reported that the presence of fluoride in pit and fissure sealant had a greater effect on remineralization and on improving mineral content of the demineralized enamel surface [24]. However, it was noticed that the effect of bioactive sealant on Ca mineral regain/recovery was higher than fluoride sealant. This could be related to the nature of the resin material since another study shed the light on the effect of matrix of resinous sealants on remineralization. They concluded that the matrix hindered remineralization as it is hydrophilic making fluoride release more difficult [25].

Nevertheless, neither bioactive sealant nor fluoride sealant was able to totally restore the Ca/P ratio to its initial baseline measurements of sound HA (1.25). This could be justified by conducting the current study in a fixed environment and not as viable as the oral cavity. The pH cycle by itself would not be sufficient to restore the lost minerals and rather another process is needed to repair the damaged enamel. Thus, the negative control group showed a lower remineralizing capacity than any of the treated surfaces by either material which is in agreement with the findings reported by Lobo et al. [26].

Unexpectedly P ions decreased after remineralization with both bioactive and fluoride sealants. These findings were consistent with the study conducted by Salman et al. [27] where they used fluoride varnish and CPP-ACP varnish on primary teeth. They found that a great reduction in phosphate content in enamel occurred after remineralization with the experimented materials. Their explanation depended on the sample selection as their study was conducted on primary enamel with its different characteristics than that of permanent enamel. In our study, the decrease in P ions could be attributed to a possible shortcoming in the experiment. The pH cycle involves that enamel is subjected to a series of demineralisation and remineralisation cycles to mimic the dynamics of mineral loss/regain [20, 28]. Probably, it was necessary to add a recharging solution during the pH cycle. Several studies advocated the use of such a solution to increase the pooling of ions [29]. This would have facilitated their uptake by the sealant to recharge it with ions. This in turn would have reflected on incorporating minerals onto the enamel surface [30]. It was markedly noticed that percentage decrease of P ions from demineralization to remineralization was significantly lower with fluoride sealant than with bioactive sealant. This was noticed to a lesser degree with bioactive sealant probably because of the increased availability of ions within the microcapsules allowing more availability of P ions to be integrated within the enamel surface. Nevertheless, P ions recovery percentage was higher with fluoride sealant more than with bioactive sealant although not being statistically significant.

Demineralized surfaces as examined by SEM, confirmed severe mineral loss by the presence of gaps among enamel crystals and surface porosity. This was consistent with the results of SEM that confirmed minerals gain and an attained improvement in the enamel surface characteristics for both bioactive sealant and fluoride sealant but with more acceptable results in surface uniformity with the bioactive pit and fissure sealant [31]. This might be attributed to the microcapsule semipermeable structure of bioactive sealant. The porosity of the resin material allowed ions to move through in both directions: from the microcapsules to increase the concentration at the tooth/sealant interface, and into the microcapsules to fill them up with ions [19] Moreover, the presence of white and granular zones at the tooth/sealant interface were confirmed for both groups and was considered positive for remineralization. These zones were resultant from the precipitation of Ca, P and F into HA in these areas. These findings were in accordance with Park et al., [15] and Choudhary et al. [23]. Both studies concluded that these zones were remineralization zones because there were no similar signs in the control group. Another study detected granular and globular zones when CPP- ACP was used on eroded enamel. They also regarded these zones as areas of remineralization [32]. However, several studies have shown conflicting results regarding the remineralization potential of the fluoride pit and fissure sealants as compared to non-fluoridated ones [24, 26]. The negative control group showed complete lack of any white zone along the interface. There were multiple irregularities detected on the surface. This was consistent with the observations noted by Ganesh and Tandon [33].

In summation, both materials bioactive sealant and fluoride sealant, compared to the control group, have the ability to enhance remineralization. However, although it was not statistically significant but bioactive sealant showed better results. Consequently, the null hypothesis was not rejected as both *bioactive and fluoride sealant proved to have good and similar remineralizing potential on demineralized enamel surface, when measured by the EDX and when interpreted by the SEM.*

### Limitations

This study might be underpowered, where a convenient sample with a limited number of teeth was used. This was because the study was conducted during COVID-19 outbreak that made the availability of a higher number of extracted teeth with the required criteria not possible. Additionally, being an in vitro experiment was another limitation of the study as it may not accurately reflect clinical conditions and the behavior of the material in vivo may become different. Moreover, the study did not take into consideration the use of conventional resin-based pit and fissure sealant as a control group which could have yielded better results than the negative control group that was used.

## Conclusions

Within the obtained results and within the limitations of the study, it could be concluded that incorporating bioactive material into pit and fissure sealant through microcapsules provided better results than incorporating fluoride by enhancing the biological process of remineralization.

### Recommendations

It is recommended that; more investigations are needed to explore the effect of microcapsules on the material’s physical properties. Nevertheless, more evaluations of the microstructures of regenerated subsurface enamel are needed to display the features of restored enamel. For more confirmation of the remineralizing effect of bioactive sealants, other re/demineralization assessment techniques such as microhardness or field emission scanning electron microscopy could be used in future studies. Moreover, comparing different types of bioactive fissure sealants; according to their mode of bioavailability, with fluoridated sealant and with a non-bioactive, non-fluoridated is recommended in further studies to evaluate the highest capacity of remineralization through the different minerals’ bioactivity. Hence, further in vivo studies on larger numbers of teeth would be beneficial to verify and confirm the efficacy of different bioactive pit and fissure sealants.

### Clinical significance

- Minimal invasive dentistry encourages the use of materials that would stop any caries lesion from progressing. It is even more beneficial if this material might regress early incipient lesions as well.
- Dealing with the child patient may be difficult at times. This necessitates the use of materials that would decrease the need in the future for operative procedures to restore their teeth.
- Bioactive sealant seems like a promising material to be used with pediatric patients fulfilling these goals.

## Data Availability

The datasets generated and analyzed during the current study are not publicly available but are available from the corresponding author on reasonable request.

## Abbreviations

EDX: Energy dispersive X-ray spectrometer
SEM: Scanning electron microscopy
HA: Hydroxyapatite
Ca: Calcium
P: Phosphate
SPSS: Statistical package for social sciences

## References

[1] James SL, Abate D, Abate KH, Abay SM, Abbafati C, Abbasi N, Abbastabar H, Abd-Allah F, Abdela J, Abdelalim A (2017). Global, regional, and national incidence, prevalence, and years lived with disability for 354 diseases and injuries for 195 countries and territories, a systematic analysis for the Global Burden of Disease Study 2017. Lancet.

[2] Mann A, Dickinson M (2006). Nanomechanics, chemistry and structure at the enamel surface. Monogr Oral Sci.

[3] Chen H, Tang Z, Liu J, Sun K, Chang SR, Peters MC, Mansfield JF, Czajka-Jakubowska A, Clarkson BH (2006). Acellular synthesis of a human enamel-like microstructure. Adv Mater.

[4] Xiao Z, Que K, Wang H, An R, Chen Z, Qiu Z, Lin M, Song J, Yang J, Lu D (2017). Rapid biomimetic remineralization of the demineralized enamel surface using nano-particles of amorphous calcium phosphate guided by chimaeric peptides. Dent Mater.

[5] Kervanto-Seppälä S, Pietilä I, Meurman JH, Kerosuo E (2009). Pit and fissure sealants in dental public health–application criteria and general policy in Finland. BMC Oral Health.

[6] Wright JT, Crall JJ, Fontana M, Gillette EJ, Nový BB, Dhar V, Donly K, Hewlett ER, Quinonez RB, Chaffin J (2016). Evidence-based clinical practice guideline for the use of pit-and-fissure sealants: a report of the American Dental Association and the American Academy of Pediatric Dentistry. J Am Dent Assoc.

[7] Griffin SO, Gray SK, Malvitz DM, Gooch BF (2009). Caries risk in formerly sealed teeth. J Am Dent Assoc.

[8] Naaman R, El-Housseiny AA, Alamoudi N (2017). The use of pit and fissure sealants—a literature review. Dent J.

[9] Schwendicke F, Frencken JE, Bjørndal L, Maltz M, Manton DJ, Ricketts D, Van Landuyt K, Banerjee A, Campus G, Doméjean S (2016). Managing carious lesions: consensus recommendations on carious tissue removal. Adv Dent Res.

[10] Hill RG, Brauer DS (2011). Predicting the bioactivity of glasses using the network connectivity or split network models. J Non-Cryst Solids.

[11] Jefferies S (2014). Bioactive and biomimetic restorative materials: a comprehensive review. Part II. J Esthetic Restor Dent.

[12] BioCoat® Pit and Fissure Sealant | Premier Dental (premierdentalco.com) https://www.premierdentalco.com/product/hygienepreventative/pit-and-fissure-sealant/biocoat/. Last accessed March 2022

[13] Association GAotWM (2014). World Medical Association Declaration of Helsinki: ethical principles for medical research involving human subjects. J Am College Dent.

[14] Ten Cate J, Duijsters P (1982). Alternating demineralization and remineralization of artificial enamel lesions. Caries Res.

[15] Park SW, Lee YK, Kim YU, Kim MC, Kim KN, Choi B, Choi H (2005). The effect of hydroxyapatite on the remineralization of dental fissure sealant. Key Eng Mater.

[16] Amaechi BT, van Loveren C (2013). Fluorides and non-fluoride remineralization systems. Monogr Oral Sci.

[17] Burbank BD, Cooper RL, Kava A, Hartjes JM, McHale WA, Latta MA, Gross SM (2017). Ion release and in vitro enamel fluoride uptake associated with pit and fissure sealants containing microencapsulated remineralizing agents. Am J Dent.

[18] Salas-López EK, Pierdant-Pérez M, Hernández-Sierra JF, Ruíz F, Mandeville P, Pozos-Guillén AJ (2017). Effect of silver nanoparticle-added pit and fissure sealant in the prevention of dental caries in children. J Clin Pediatric Dent.

[19] Biocoat guide [https://www.premierdentalco.com/wp-content/uploads/2017/04/biocoat_technical-guide_PART1_R1.pdf] Last accessed April 2022

[20] Lippert F, Juthani K (2015). Fluoride dose-response of human and bovine enamel artificial caries lesions under pH-cycling conditions. Clin Oral investig.

[21] Tantbirojn D, Huang A, Ericson M, Poolthong S (2008). Change in surface hardness of enamel by a cola drink and a CPP–ACP paste. J Dent.

[22] Besinis A, van Noort R, Martin N (2016). The use of acetone to enhance the infiltration of HA nanoparticles into a demineralized dentin collagen matrix. Dent Mater.

[23] Choudhary P, Tandon S, Ganesh M, Mehra A (2012). Evaluation of the remineralization potential of amorphous calcium phosphate and fluoride containing pit and fissure sealants using scanning electron microscopy. Indian J Dent Res.

[24] Salar DV, Garcia-Godoy F, Flaitz CM, Hicks MJ (2007). Potential inhibition of demineralization in vitro by fluoride-releasing sealants. J Am Dent Assoc.

[25] Preston AJ, Agalamanyi EA, Higham SM, Mair LH (2003). The recharge of esthetic dental restorative materials with fluoride in vitro—two years' results. Dent Mater.

[26] Lobo MM, Pecharki GD, Tengan C, da Silva DD, da Silva Tagliaferro EP, Napimoga MH (2005). Fluoride-releasing capacity and cariostatic effect provided by sealants. J Oral Sci.

[27] Salman NR, ElTekeya M, Bakry N, Omar SS, El Tantawi M (2019). Comparison of remineralization by fluoride varnishes with and without casein phosphopeptide amorphous calcium phosphate in primary teeth. Acta Odontol Scand.

[28] Buzalaf MAR, Hannas AR, Magalhães AC, Rios D, Honório HM, Delbem ACB (2010). pH-cycling models for in vitro evaluation of the efficacy of fluoridated dentifrices for caries control: strengths and limitations. J Appl Oral Sci.

[29] Memarpour M, Afzali Baghdadabadi N, Rafiee A, Vossoughi M (2020). Ion release and recharge from a fissure sealant containing amorphous calcium phosphate. Plos one.

[30] Garcia IM, Balhaddad AA, Aljuboori N, Ibrahim MS, Mokeem L, Ogubunka A, Collares FM, de Melo MAS (2021). Wear behavior and surface quality of dental bioactive ions-releasing resins under simulated chewing conditions. Front Oral Health.

[31] Elsherbini MS (2020). Assessment of remineralization potential of Theobromine and Sodium Fluoride gels on Artificial Caries like lesions. Brazil Dent Sci.

[32] Ramalingam L, Messer L, Reynolds E (2005). Adding casein phosphopeptide-amorphous calcium phosphate to sports drinks to eliminate in vitro erosion. Pediatr Dent.

[33] Ganesh M, Tandon S (2007). Clinical evaluation of FUJI VII sealant material. J Clin Pediatric Dent.

